# Efficacy and harms of remdesivir for the treatment of COVID-19: A systematic review and meta-analysis

**DOI:** 10.1371/journal.pone.0243705

**Published:** 2020-12-10

**Authors:** Alejandro Piscoya, Luis F. Ng-Sueng, Angela Parra del Riego, Renato Cerna-Viacava, Vinay Pasupuleti, Yuani M. Roman, Priyaleela Thota, C. Michael White, Adrian V. Hernandez

**Affiliations:** 1 Unidad de Revisiones Sistemáticas y Meta-análisis (URSIGET), Vicerrectorado de Investigación, Universidad San Ignacio de Loyola (USIL), Lima, Peru; 2 Hospital Guillermo Kaelin de La Fuente, Lima, Peru; 3 Department of Internal Medicine, Henry Ford Hospital, Detroit, Michigan, United States of America; 4 Escuela de Medicina, Universidad Peruana de Ciencias Aplicadas (UPC), Lima, Peru; 5 MedErgy Health Group Inc. Yardley, Pennsylvania, United States of America; 6 Health Outcomes, Policy, and Evidence Synthesis (HOPES) Group, University of Connecticut School of Pharmacy, Storrs, Connecticut, United States of America; 7 Department of Research Administration, Hartford Hospital. Hartford, Connecticut, United States of America; 8 Hemex Health Inc. Portland, Oregon, United States of America; All India Institute of Medical Sciences, Bhopal, INDIA

## Abstract

**Background:**

Efficacy and safety of treatments for hospitalized COVID-19 are uncertain. We systematically reviewed efficacy and safety of remdesivir for the treatment of COVID-19.

**Methods:**

Studies evaluating remdesivir in adults with hospitalized COVID-19 were searched in several engines until August 21, 2020. Primary outcomes included all-cause mortality, clinical improvement or recovery, need for invasive ventilation, and serious adverse events (SAEs). Inverse variance random effects meta-analyses were performed.

**Results:**

We included four randomized controlled trials (RCTs) (n = 2296) [two vs. placebo (n = 1299) and two comparing 5-day vs. 10-day regimens (n = 997)], and two case series (n = 88). Studies used intravenous remdesivir 200mg the first day and 100mg for four or nine more days. One RCT (n = 236) was stopped early due to AEs; the other three RCTs reported outcomes between 11 and 15 days. Time to recovery was decreased by 4 days with remdesivir vs. placebo in one RCT (n = 1063), and by 0.8 days with 5-days vs. 10-days of therapy in another RCT (n = 397). Clinical improvement was better for 5-days regimen vs. standard of care in one RCT (n = 600). Remdesivir did not decrease all-cause mortality (RR 0.71, 95%CI 0.39 to 1.28, I^2^ = 43%) and need for invasive ventilation (RR 0.57, 95%CI 0.23 to 1.42, I^2^ = 60%) vs. placebo at 14 days but had fewer SAEs; 5-day decreased need for invasive ventilation and SAEs vs. 10-day in one RCT (n = 397). No differences in all-cause mortality or SAEs were seen among 5-day, 10-day and standard of care. There were some concerns of bias to high risk of bias in RCTs. Heterogeneity between studies could be due to different severities of disease, days of therapy before outcome determination, and how ordinal data was analyzed.

**Conclusions:**

There is paucity of adequately powered and fully reported RCTs evaluating effects of remdesivir in hospitalized COVID-19 patients. Until stronger evidence emerges, we cannot conclude that remdesivir is efficacious for treating COVID-19.

## Introduction

Worldwide, ~50 million patients have been infected with Coronavirus Disease 2019 (COVID-19), resulting in over 1.2 million deaths [1]. Older populations with obesity, hypertension, diabetes, and chronic kidney disease have a poorer prognosis when infected [2]. In the most severely ill COVID-19 patients, corticosteroid therapy has been shown to prolong survival but no other drugs have demonstrated efficacy in randomized controlled trials (RCTs) with a reasonable safety profile [3–5].

Remdesivir inhibits ribonucleic acid (RNA) polymerase limiting viral replication [6, 7]. It was originally developed to treat Ebola but promising in vitro effects were not translated into acceptable clinical efficacy. Remdesivir provides antiviral effects on coronaviruses in vitro and early initiation of therapy significantly reduced pulmonary damage in monkeys infected with COVID-19 [6, 8]. Remdesivir received Food and Drug Administration (FDA) Emergency Use Authorization (EUA) on May 1^st^ 2020 and was approved on October 22, 2020 [9]. We systematically evaluated the human studies assessing the efficacy and safety of remdesivir for the treatment of COVID-19.

## Materials and methods

### Search strategy and selection criteria

We performed a systematic review of RCTs and observational studies evaluating the effects of remdesivir in adult hospitalized COVID-19 patients with pneumonia and/or respiratory insufficiency. We searched for studies in PubMed, Web of Science, Scopus, Embase and the Cochrane Library, and medRxiv.org and for ongoing RCTs in www.ClinicalTrials.gov, www.who.int/ictrp/about/en/, and www.clinicaltrialsregister.eu/. Databases were searched on May 5^th^, 2020 and updated on June 5th, 2020 and August 21, 2020.

Search strategies were adjusted for each engine using the keywords: remdesivir AND (COVID-19 OR coronavirus OR coronavirus disease OR coronavirus disease-19 OR severe acute respiratory syndrome OR SARS-CoV-2) with no limitations for time or language. The PubMed strategy is included in the S1 File. Included studies in our search involved case series, cohorts and RCTs that specified at least one efficacy or harm outcome. We excluded studies with hepatitis B or HIV coinfection patients.

### Data extraction

Three reviewers (VP, AP, LFN-S) collected records in www.myendnoteweb.com. Two independent reviewers (APdR, RC-V) assessed titles and abstracts for eligibility according to the inclusion and exclusion criteria. Discrepancies were resolved by discussion. Three reviewers (LFN-S, APdR, RC-V) assessed full-text articles of selected studies. If two reviewers were unable to reach consensus, they consulted a third review author (AP). Three independent reviewers (LFN-S, APdR, RC-V) extracted data with disagreements resolved by a third reviewer (AP). Extracted information included: study authors, year of publication, study design, number of patients, country, median age, proportion of males, comorbidities (obesity, hypertension, diabetes, coronary artery disease (CAD), chronic kidney disease, chronic obstructive pulmonary disease), PCR method for COVID-19 diagnosis, remdesivir dose and duration, concomitant treatments for both arms, primary outcomes per arm, and secondary outcomes per arm.

### Outcomes

Primary outcomes were: all-cause mortality, clinical improvement (e.g. 2-point reduction in a 6-point ordinal severity scale), time to recovery (defined as first day, during 28-day enrollment, on which a patient satisfied categories 1, 2, or 3 of an 8-point ordinal scale), need for invasive ventilation (mechanical [MV], extracorporeal membrane oxygenation [ECMO]), and serious adverse events (SAE). Secondary outcomes were length of hospital stay, components of ordinal severity scales, radiological progression of pneumonia, and adverse events (AE).

### Assessment of risk of bias

Assessment of risk of bias was performed independently by two investigators (VP, AVH) using the Cochrane RoB 2.0 tool [10] for RCTs. We planned to independently use the ROBINS-I tool [11] for cohort studies but no observational studies were found. A third reviewer (AP) resolved discrepancies when needed.

### Statistical analyses

We reported our systematic review according to 2009 PRISMA guidelines [12]. Effects of remdesivir on outcomes from individual studies were reported as hazard ratio (HR) or risk difference (RD) or relative risk (RR) for dichotomous outcomes and mean differences (MD) for continuous outcomes, each with 95% confidence intervals (95% CIs). Inverse variance random effect meta-analyses were performed when outcome data was available for at least two RCTs judged to have homogeneous study characteristics. Between study variance tau^2^ was calculated with the Paule-Mandel method. Effects of meta-analyses were reported as relative risks (RR) and their 95%CIs, and heterogeneity of effects among studies was quantified with the I^2^ statistic (an I^2^>60% means high heterogeneity of effects). R 3.5.1 (www.r-project.org) was used for meta-analyses. The quality or certainty of evidence was evaluated using the GRADE methodology, which covers 5 items: risk of bias, inconsistency, indirectness, imprecision, and publication bias [13]. Quality of evidence was evaluated per specific comparison and per outcome, and described in summary of findings (SoF) tables; GRADEpro GDT was used to create SoF tables [14].

## Results

**Fig 1:**
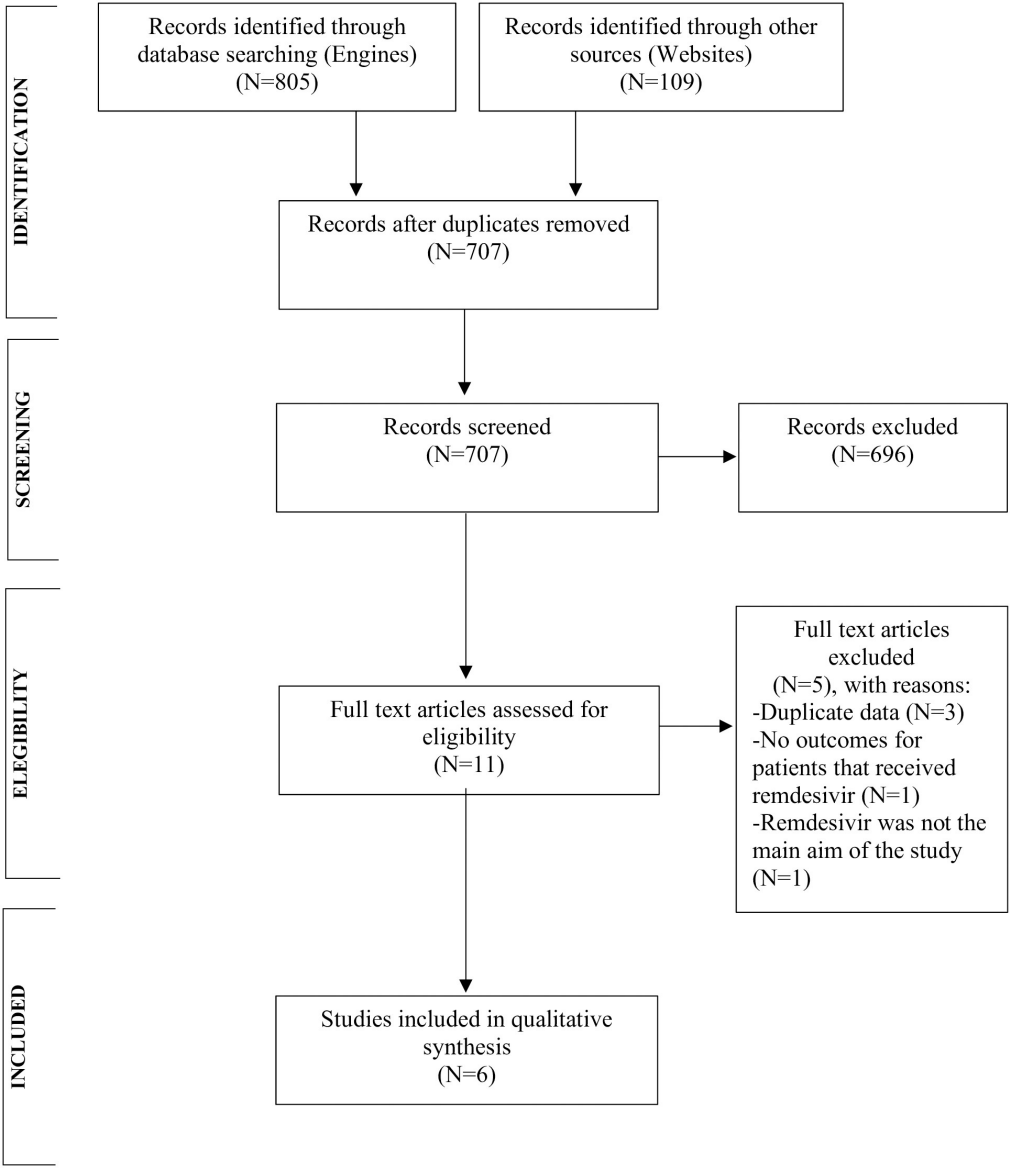
Flowchart of study selection.

From our searches, 553 records were screened for eligibility and eleven full-text articles were assessed (Fig 1). One RCT planned in China (NCT04252664) was suspended on April 15^th^, 2020. Five articles were excluded leaving two placebo-controlled RCTs (n = 1300), two RCTs comparing 5-day vs. 10-day regimens of remdesivir with or without a standard of care arm (n = 997), and two case series (n = 88) [15–20].

Five of the six included studies had patients with similar baseline characteristics including severe or life-threatening patients with an oxygen saturation <94%. The Spinner et al. trial was unique in that it evaluated patients with mild to moderate severity of disease. The sample sizes ranged from 35 to 53 participants across case series, and 236 to 1063 across trials. Most of these studies compared intravenous (IV) remdesivir 200 mg as a loading dose and then 100 mg daily for 10 days vs. placebo or standard of care. However, Goldman et al. provided the 200mg loading dose to all participants and compared the 100mg daily dose for10 days vs. 5 days. The follow-up timeframe was 28 days in both case series and ranged between 11 and 29 days across trials.

The description and demographic information of the included studies are shown in Table 1. The median age of patients was between 63 to 64 years across case series and 58 to 66 years across trials. There was a predominance of male gender ranging between 56.3% to 75.4% across studies and comorbidities such as hypertension (24.5% to 50%) and diabetes (8.6% to 30.6%) varied between trials. Similar concomitant treatments were given in the trials such as corticosteroids, antivirals, antibiotics, and support therapy according to each hospital policy.

**Table 1:** Description of characteristics of included studies.

	Grein et al. [17]	Antinori et al. [18]	Wang et al. [15]		Beigel et al. ACTT-1 [16]		Goldman et al. [19]		Spinner et al. SIMPLE [20]	
**Design**	Case series	Case series	RCT		RCT		RCT		RCT	
**Country(ies)**	United States, Japan, Italy, Austria, France, Germany, Netherlands, Spain, Canada	Italy	China		Denmark, Germany, Greece, Japan, Korea, Mexico, Singapore, Spain, United Kingdom, United States		United States, Italy, Spain, Germany, Hong Kong, Singapore, South Korea, Taiwan		United States, China, France, Germany, Hong Kong, Italy, Japan, Korea, the Netherlands, Singapore, Spain, Sweden, Switzerland, Taiwan and the United Kingdom	
**Population**	Adults with COVID-19 RT-PCR +, O_2_ sat <94%, within 15 days of symptom onset.	Adults with COVID 19 RT-PCT +, O2 sat <94% or mechanically ventilated or NEWS2 score ≥4, within 10 days of symptom onset.	Adults with COVID-19 RT-PCR + infection, pneumonia in chest imaging, sat O_2_ < 94% or PaO_2_/FiO_2_ < 300mmHg, within 12 days of symptom onset.		Patients ≥ 18 years old with COVID-19 PCR + infection (72hrs prior randomization or > 72hrs if disease consistent with COVID-19), radiographic infiltrates in chest imaging or sat O_2_ < 94% or requiring supplemental O_2_ or requiring mechanical ventilation. Within 13 days of symptom onset.		Patients ≥12 years old, PCR + COVID-19 confirmed infection, sat O_2_ ≤94% and radiologic image of pneumonia. Within 12 days of symptom onset.		Patients ≥12 years old with SARS-CoV-2-confirmed moderate COVID-19 infection and evidence of pneumonia without reduced oxygen levels (sat O_2_ >94%).	
**Exclusion criteria**	No exclusion criteria specified in any segment of the manuscript, supplement or protocol.	ALT or AST >5 times the upper limit of the normal range; creatinine clearance <30 mL/min	Pregnancy or breastfeeding; hepatic cirrhosis; ALT or AST more than five >5 times the upper normal of limit; known severe renal impairment (estimated Glomerular filtration rate <30mL/min per 1.73 m^2^ or receipt of continuous RRT, hemodialysis, or peritoneal dialysis: possibility of transfer to a non-study hospital within 72hr; enrolment into an investigational treatment study for COVID-19 in the 30 days before screening.		ALT or AST > 5 times the upper limit of normal; stage 4 severe CKD or requiring dialysis (i.e. eGFR < 30); pregnancy or breastfeeding; anticipated transfer to another hospital which is not a study site within 72 hours; allergy to any study medication		ALT or AST > 5 times the upper limit of normal, estimated creatinine clearance of less than 50 ml per minute (by the Cockcroft–Gault formula), receiving concurrent treatment (within 24 hours before the start of trial treatment) with other agents with putative activity against Covid-19.		ALT or AST >5 times the upper limit of the normal range; creatinine clearance <50mL/min	
**Sample size**	53	35	236		1063		397		600	
**Reported primary outcome**	Proportion of clinical improvement (live discharge from the hospital, a decrease of at least two points from baseline on the modified 6-point ordinal severity scale)	Change in patient hospitalization status using a 7-point ordinal severity scale (1, not hospitalized, capable of resuming normal activities; 2, not hospitalized but unable to resume normal activities; 3, hospitalized, not requiring O_2_ supplementation; 4, hospitalized and requiring O_2_ therapy; 5, hospitalized and requiring high flow nasal O_2_ therapy, non-invasive MV, or both; 6, ICU hospitalization, requiring invasive MV or ECMO, or both; 7, death.	Time to clinical improvement within 28 days or discharged alive from hospital. Clinical improvement: decline of two points using a 6-point ordinal severity scale (6 = death, 5 = hospital admission for ECMO or MV, 4 = hospital admission for non-invasive ventilation or high-flow O_2_ therapy, 3 = hospital admission for O_2_ therapy (low flow O_2_ therapy), 2 = hospital admission without O_2_ therapy, and 1 = discharged or having reached clinical recovery).		Time to recovery at 29 days (Preliminary report at day 15). Recovery was defined as the first day during the 28 days after enrollment on which a patient satisfied category 1, 2 or 3 in the 8-point category scale (1, not hospitalized, no limitations of activities; 2, not hospitalized, limitation of activities, home O_2_ requirement, or both; 3, hospitalized, not requiring supplemental O_2_ and no longer requiring ongoing medical care (used if hospitalization was extended for infection-control reasons); 4, hospitalized, not requiring supplemental O_2_ but requiring ongoing medical care (Covid-19– related or other medical conditions); 5, hospitalized, requiring any supplemental O_2_; 6, hospitalized, requiring noninvasive ventilation or use of high-flow oxygen devices; 7, hospitalized, receiving invasive MV or ECMO; and 8, death).		Clinical status on day 14, assessed on a 7-point ordinal severity scale (1, death; 2, hospitalized, receiving invasive MV or ECMO; 3, hospitalized, receiving non-invasive ventilation or high-flow O_2_ devices; 4, hospitalized, requiring low-flow supplemental O_2_; 5, hospitalized, not requiring supplemental O_2_ but receiving ongoing medical care (related or not related to Covid-19); 6, hospitalized, requiring neither supplemental O_2_ nor ongoing medical care (other than that specified in the protocol for remdesivir administration); 7, not hospitalized)		Clinical status on day 11, assessed on a 7-point ordinal severity scale; including: ≥ 2-point improvement in ordinal scale, ≥1-point improvement in ordinal scale, Requiring any oxygen support, ≥ 1-point worsening in ordinal scale, and death.	
**Intervention**	Remdesivir 200mg loading dose day 1°, then 100mg once daily for a up to 10 days	Remdesivir 200mg loading dose day 1°, then 100mg once daily for a up to 10 days	Remdesivir 200mg loading dose day 1°, then 100mg once daily for a up to 10 days		Remdesivir 200mg loading dose day 1°, then 100mg once daily for a up to 10 days		Remdesivir 200mg loading dose day 1°, then 100mg once daily for a up to 5 days		Remdesivir 200mg loading dose day 1°, then 100mg once daily for a up to 10 days (5-day course or 10-day course)	
**Comparator**	None	None	Placebo (blinded)		Placebo (blinded)		Remdesivir 200mg loading dose day 1°, then 100mg once daily for a up to 10 days		Standard of care	
**Follow up time**	28 days	28 days	28 days		29 days		14 days		11 days	
**Concomitant treatment**	Support therapy	Hydroxychloroquine	Support therapy, steroids, lopinavir/ritonavir, interferon alfa 2b		Support therapy and other treatments indicated in written hospital policy		Support therapy		Standard of care	
	Intervention	Intervention	Intervention	Control	Intervention	Control	Intervention	Control	5-day course	SOC
**Age (median, IQR)**	64 (48–71)	63 (51–69)	66 (57–73)	64 (53–70)	58.6 (14.6)	59.2 (15.4)	61 (50–69)	62 (50–71)	58 (46–66)	57 (45–66)
**Male (%)**	40 (75.4)	26 (74.3)	89 (56.3)	51 (65.3)	352 (65.1)	332 (63.6)	120 (60)	33 (67.5)	114 (60.0)	125 (62.5)
**Comorbidities (n, %)**										
**-Hypertension**	13 (24.5)	12 (34.3)	72 (45.6)	30 (38.4)	231 (49.3)	229 (49.9)	100 (50)	98 (49.2)	82 (42.9)	81 (40.5)
**-DM**	9 (17)	2 (8.6)	40 (25.3)	16 (20.5)	144 (30.6)	131 (28.7)	47 (23.5)	43 (21.8)	71 (37.0)	76 (38.0)
**-Hyperlipidemia**	6 (11.3)	NA	15 (9.5)	2 (2.5)	NA	NA	NA	NA	NA	NA
**-CHD**	NA	NA	NA	NA	61 (13)	46 (8.8)	40 (20)	49 (24.9)	111 (58.1)	107 (53.5)
**-Asthma**	6 (11.3)	NA	NA	NA	59 (12.6)	47 (10.3)	27 (13.5)	22 (11.2)	22 (11.5)	28 (14.0)
**6-point ordinal severity scale at baseline (n, %)**										
	*	†	§	§	‖	‖	¶	¶	¶	¶
**-Category 1**	0 (0)	0 (0)	0 (0)	0 (0)	0 (0)	0 (0)	0 (0)	0 (0)	0 (0)	0 (0)
**-Category 2**	2 (3.8)	1 (2.9)	0 (0)	3 (3.8)	67 (12.4)	60 (11.5)	34 (17)	21 (10.7)	160 (83.8)	162 (81.0)
**-Category 3**	10 (18.9)	2 (5.7)	129 (81.6)	65 (83.3)	222 (41)	199 (38.1)	113 (56.5)	113 (54.3)	29 (15.2)	36 (18.0)
**-Category 4**	7 (13.2)	16 (45.7)	28 (17.7)	9 (11.5)	98 (18.1)	99 (19)	49 (24.5)	60 (30.5)	2 (1.0)	2 (1.0)
**-Category 5**	34 (64.1)	16 (45.7)	0 (0)	1 (1.3)	125 (23.1)	147 (28.2)	4 (2.0)	9 (4.6)	0 (0)	0 (0)
**-Category 6**	0 (0)	0 (0)	1 (0.7)	0	0 (0)	0 (0)	0 (0)	0 (0)		
**6-point ordinal severity scale after treatment (n, %)**										
	*	†	§	§	‖	‖	¶	¶	¶	¶
**-Category 1**	25 (47.2)	20 (57.1)	39 (25.4)	18 (23)	257 (59.2)	203 (49.5)	120 (60.0)	103 (53.3)	134 (70.2)	120 (60.0)
**-Category 2**	8 (15.1)	1(2.9)	21 (13.7)	10 (12.8)	34 (7.8)	26 (6.4)	20 (10.0)	16 (8.1)	45 (23.6)	54 (27.0)
**-Category 3**	0 (0)	1(2.9)	61 (39.9)	28 (35.9)	34 (7.8)	40 (9.8)	19 (9.5)	14 (7.1)	7 (3.7)	11 (5.5)
**-Category 4**	3 (5.7)	1(2.9)	13 (8.6)	8 (10.3)	16 (3.7)	14 (3.4)	9 (4.5)	10 (5.1)	5 (2.6)	7 (3.5)
**-Category 5**	10 (18.9)	3 (8.5)	4 (2.6)	7 (9.0)	60 (13.8)	72 (17.6)	16 (8.0)	33 (16.7)	0 (0)	4 (2.0)
**-Category 6**	7 (13.2)	9 (25.7)	15 (9.8)	7 (9.0)	33 (7.6)	55 (13.4)	16 (8).0	21 (10.7)		
**Clinical improvement/recovery at day 28 (n, %)**							#	#	**	**
	36 (67.9)	22 (62.8) ‡	103 (65.0)	45 (58)	NA	NA	129 (64.5)	107 (54.3)	134 (70.2)	121 (60.5)

IQR: Interquartile range; CAD: Coronary artery disease; NA: Not applicable; O_2_: Oxygen; ICU: Intensive care unit; MV: Mechanical ventilation; ECMO: Extracorporeal membrane oxygenation; ALT: alanine aminotransferase; AST: aspartate aminotransferase; CKD: chronic kidney disease; RRT: renal replacement therapy.

*After treatment: day 15.

^†^7-category ordinal scale in this study have been adapted to this table. We combined category 1 and 2 to fit the 6-point ordinal severity scale. After treatment: day 28.

^‡^ The study main outcome was to assess any change in hospitalization status according to the 7-point ordinal severity scale. For the purposes of this table, we have considered the patients that had a positive change in clinical status at day 28^th^. In this scenario, 15 patients out the Infectious diseases wards and 7 patients in the intensive care unit had clinically changed to a better clinical status.

^§^ After treatment: day 14.

^‖^8-category ordinal scale in this study has been adapted to this table. We combined category 1 and 2; and 3 and 4 to fit the 6-point ordinal severity scale. After treatment: day 15.

^¶^7-category ordinal scale in this study has been adapted to this table. We reversed the categories since the scale was inverted as compared with the other studies. We combined their category 6 and 5 as hospitalized but not requiring supplemental oxygen. The rest of the categories are the same but inverted. After treatment day 14.

^#^ At day 14.

** Clinical improvement defined as improvement in 2 or more points in ordinal severity scale, at day 11.

### Description of studies

#### Wang et al. RCT

The RCT by Wang et al. (NCT04257656) assessed IV remdesivir 200 mg on day one, followed by 100mg IV once-daily for nine more days vs. placebo in adults with RT-PCR confirmed SARS-CoV-2 infection, pneumonia, and respiratory insufficiency in Wuhan, China (Table 1) [15]. The primary outcome was the time to clinical improvement within 28 days after randomization or time discharged alive from the hospital, whichever came first. Clinical improvement was defined as a decline of two points using a 6-point ordinal severity scale (Table 1). This scale was modified from a 7-point ordinal severity scale used by the COVID-19 lopinavir/ritonavir RCT by Cao et al. [21], which has been used in previous influenza studies by Wang et al. [22] and recommended by the World Health Organization (WHO) R&D Blueprint expert group [23].

Due to higher occurrence of AEs leading to drug discontinuation vs. placebo (12% vs 5%), the trial was stopped early, with 236 patients and a statistical power of 56%. Authors mentioned that they followed specific a priori termination criteria, but these are not available in the protocol [24]. Some imbalances existed at enrollment between arms, including more patients with hypertension, diabetes, or CAD in the remdesivir arm. Also, more patients in the control group had been symptomatic for ≤10 days at the time the intervention was started.

#### Beigel et al. Adaptive COVID-19 Treatment Trial (ACTT-1)

The multinational Beigel et al. ACTT-1 RCT (NCT04280705) evaluated remdesivir 200 mg IV on day 1, followed by a 100mg IV once-daily for nine more days vs. placebo in adults with RT-PCR confirmed SARS-CoV-2 infection, pneumonia, and respiratory insufficiency (Table 1) [16, 25]. According to clinicaltrials.gov, on February 20^th^ 2020, its primary outcome was supposed to be percentage of each severity category at 15 days on the 7-point ordinal scale by Cao et al. [21]. On March 20^th^ 2020, the primary outcome was changed to a new 8-point ordinal severity scale, in which a subdivision into two groups was made on hospitalized patients (Table 1).

On March 22^nd^ 2020, blinded statisticians recommended again changing the outcome to time to recovery, defined as the first day during the 28 days satisfying category 1, 2 or 3 of the 8-point scale [16]. This trial was stopped on April 29^th^ 2020, as its safety monitoring board determined the primary efficacy endpoint had been achieved [25, 26]. At that moment, 1063 had been recruited with 482 recoveries and 81 deaths entered to the database [16]. No substantial imbalances in baseline characteristics were observed between the remdesivir and placebo arms.

#### Goldman et al. GS-US-540-5773 RCT

The multinational Goldman et al. GS-US-540-5773 Gilead RCT (NCT04292899) evaluated the efficacy and safety of 5-day vs. 10-day remdesivir in patients ≥12 years-old, PCR confirmed SARS-CoV-2 infection, pneumonia and respiratory insufficiency (Table 1) [19]. The primary outcome was changed after enrollment from proportion of patients with normalization of temperature at day 14 to clinical status evaluated at 14 days by a 7-point ordinal severity scale based on the 6-point scale of Wang et al. [15] but divided their non-oxygen user hospitalized patients into two categories regarding their need for clinical care. Both groups received supportive therapy at the discretion of the clinician. Secondary outcomes were time to clinical improvement (≥2 points of ordinal scale), time to recovery (improvement from a baseline score of 2–5 to 6–7), time to modified recovery (improvement from a baseline score of 2–4 to 5–7 or from 5 to 6–7), all-cause mortality and safety.

#### Spinner et al. SIMPLE RCT

Gilead’s open-label SIMPLE trial (NCT04292730) evaluated the efficacy and safety of remdesivir 5-day or 10-day plus standard of care vs. standard of care alone in hospitalized patients with SARS-CoV-2 confirmed infection and moderate pneumonia (e.g. without reduced oxygen levels) [20]. The primary outcome was clinical status evaluated by a 7-point ordinal severity scale at day 11 and the secondary outcome was the rate of adverse events between treatment arms. Clinical improvement was defined as 2 points improvement in the 7-point ordinal scale; also, a one-point improvement was assessed as well as the requirement of oxygen support or worsening of one point in the scale.

#### Case series

The case series by Grein et al. [17] assessed 53 RT-PCR confirmed COVID-19 patients, with oxygen saturation <94% receiving a similar 10-day remdesivir regimen and follow up as placebo-controlled RCTs [15, 16]. Clinical improvement (live discharge from the hospital, a decrease of ≥2 points on a modified 6-point ordinal scale) as suggested by the WHO [23], changes in oxygen support, AEs, discharge, and deaths were recorded. Mean age was 64 years and 75% were male. About 60% had hypertension, diabetes, hyperlipidemia and asthma and most were on low flow oxygen support or invasive ventilation.

The case series by Antinori et al. evaluated 35 RT-PCR confirmed COVID-19 patients, with oxygen saturation <94% or mechanically ventilated or NEWS-2 score ≥4 [18] with similar remdesivir regimen and follow up as RCTs. They used the 7-point ordinal scale used by Cao et al. [21] Median age was 63 years, 74% male, with 9% diabetes and 34% hypertension. Eighteen patients were in the ICU and 17 in an infectious disease ward.

### Effect of remdesivir on primary outcomes

**Fig 2:**
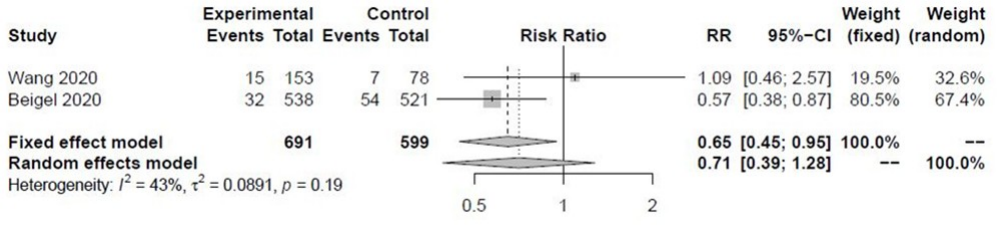
Effect of remdesivir on all-cause mortality at 14 days.

There was no significant reduction in all-cause mortality vs. placebo at 14 days (RR 0.71, 95%CI 0.39 to 1.28, I^2^ = 43%, Fig 2). There was no difference in mortality between 5-day and 10-day remdesivir arms in Goldman et al. (RR 0.75, 95%CI 0.40 to 1.40) [19]. All-cause mortality was not different between the 10-day, 5-day and SOC arms at 11 days (1%, 0%, 2%, respectively), and at 28 days (2%, 1%, 2%, respectively) in the Spinner et al. trial. [20] Grein et al. reported that 13% died [17], whereas in Antinori et al. 26% died [18].

Time to clinical improvement was not different between arms in Wang et al. (HR 1.23, 95% CI 0.87–1.75), but time to recovery was significantly shorter with remdesivir in Beigel et al. (11 days, 95%CI 9 to 12 vs. 15 days, 95% CI 13 to 19, RR for recovery 1.32; 95%CI 1.12 to 1.55) in comparison with placebo. In Goldman et al., time to recovery (HR 0.81, 95%CI 0.64 to 1.04) and time to clinical improvement (HR 0.79, 95%CI 0.61 to 1.01) were different between arms [19]. In the Spinner et al. trial, the odds of clinical improvement at day 11 was higher for 5-day (134/191 [70.2%], RD 9.7%, 95%CI 0.1% to 19.1%) but not for 10-day (126/193 [65.3%], RD 4.8%, 95%CI -5.0% to 14.4%) remdesivir vs. standard of care (121/200 [60.5%]) [20].

**Fig 3:**
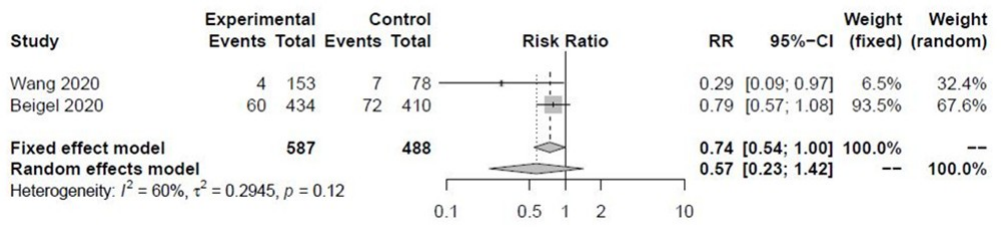
Effect of remdesivir on invasive ventilation at 14 days.

Remdesivir did not decrease the need for invasive ventilation vs. placebo at 14 days (RR 0.57, 95%CI 0.23 to 1.42, I^2^ = 60%, Fig 3). Five-days of remdesivir treatment reduced the need for invasive ventilation vs. 10-day in Goldman et al. (RR 0.48, 0.27 to 0.84) [19]. SAEs were significantly lower with remdesivir vs. placebo (RR 0.77, 95%CI 0.63 to 0.94) (S1 Fig). In Beigel et al. ACCT-1, SAEs were present in 21% vs. 27% in remdesivir and placebo, respectively [16]. The most common SAEs were acute respiratory failure, hypotension, viral pneumonia and acute kidney injury but were more frequent in the placebo arm. In Goldman et al., SAEs were more frequent in the 10-day remdesidivir arm (21% vs 35%), with respiratory failure being the most commonly seen [19]. SAEs were less frequent in the 5-day and 10-day remdesivir vs. SOC arms (4.7%, 5.2%, and 9.0%) in the Spinner et al. trial [20]. Grein et al. reported 23% of SAEs, most commonly multiple organ failure, septic shock, kidney injury and hypotension with an 8% drug discontinuation rate [17].

### Effect of remdesivir on secondary outcomes

There was no effect of remdesivir on hospitalization without oxygen or with oxygen support/non-invasive ventilation vs. placebo (S2 and S3 Figs; explanation of ordinal scale re-categorization across RCTs in S3 File). A higher proportion of patients were discharged if treated with remdesivir (S4 Fig), and there was no difference in treatment discontinuation (S5 Fig) vs. placebo. There was no difference in AEs in the two placebo-controlled RCTs (RR 0.94, 95%CI 0.81 to 1.10) (S6 Fig). AEs were similar between 5-day and 10-day remdesivir arms (71 vs 74%) [17], but higher in 5-day or 10-day arm vs. standard of care arm (51.3%, 58.5%, 46.5%) [18]. No differences were found between remdesivir vs. placebo on specific AEs such as anemia, elevated liver enzymes, hyperbilirubinemia, hypoalbuminemia, deep vein thrombosis, pulmonary embolism or renal impairment (S7–S13 Figs).

Antinori et al. reported that 43% of subjects had elevated liver enzymes, 23% had acute kidney injury, and 20% had elevated bilirubin levels [18]. Overall, 23% of AEs led to drug discontinuation [18]. Grein et al. reported that 60% of patients experienced AEs including elevated liver enzymes (23%), diarrhea (9%) and rash (8%) [17]. None of the studies documented radiological progression of pulmonary disease or viral clearance.

### Quality of evidence and risk of bias of RCTs

The quality of evidence for primary and secondary outcomes per comparison are shown in Table 2 (remdesivir 10 days vs. placebo), Table 3 (remdesivir 5 days vs. 10 days), Table 4 (remdesivir 5 days vs. standard of care), and S1 Table (remdesivir 10 days vs. standard of care). Overall quality of evidence was low or very low for all primary and secondary outcomes, mainly due to some concerns or high risk of bias among RCTs, and inconsistency and imprecision of effects. Indirectness was present when assessing the Spinner et al. trial as its population were moderate COVID-19 patients in contrast to severe COVID-19 patients in other three trials. The risk of bias analysis of four RCTs using Cochrane’s RoB 2.0. tool is shown in S14 and S15 Figs. The Wang et al. trial was judged to have some concerns due to potential confounding on the randomization process. The Beigel et al. trial was judged to have high risk of bias due to selection of the reported results, as their primary outcome was time to recovery after two previous changes. The Goldman et al. trial was judged to have serious risk of bias due to the measurement of the outcome, as they used a non-validated 7-point disease severity scale. The Spinner et al. trial was judged to have some concerns due to selection of the reported results.

**Table 2:** Summary of findings table for the comparison remdesivir 10 days vs. placebo for hospitalized, severe COVID-19.

Patient or population: hospitalized, severe COVID-19					
Setting: Hospital					
Intervention: remdesivir for 10 days					
Comparison: placebo					
Outcomes	Anticipated absolute effects* (95% CI)		Relative effect (95% CI)	No of participants (studies)	Certainty of the evidence (GRADE)
	Risk with placebo	Risk with remdesivir for 10 days			
All-cause mortality follow up: range 15 days to 28 days	10 per 100	**7 per 100** (4 to 13)	**RR 0.71** (0.39 to 1.28)	1290 (2 RCTs)	⨁◯◯◯ VERY LOW ^a^^,^^b^^,^^c^
Clinical improvement assessed with: Decline of 2 points using 6-points ordinal severity scale follow up: 28 days	58 per 100	**65 per 100** (53 to 78)	**HR 1.23** (0.87 to 1.75)	236 (1 RCT)	⨁⨁◯◯ LOW ^d^^,^^e^
Recovery assessed with: First day when patient satisfied categories 1, 2 or 3 of an 8-point ordinal severity scale follow up: 15 days	52 per 100	**69 per 100** (59 to 81)	**Rate ratio 1.32** (1.12 to 1.55)	1059 (1 RCT)	⨁⨁◯◯ LOW ^f^
Need for invasive ventilation follow up: 14 days	16 per 100	**9 per 100** (4 to 23)	**RR 0.57** (0.23 to 1.42)	1075 (2 RCTs)	⨁◯◯◯ VERY LOW ^a^^,^^g^^,^^h^
Hospitalization without oxygen follow up: 15 days	7 per 100	**9 per 100** (6 to 13)	**RR 1.18** (0.79 to 1.76)	1075 (2 RCTs)	⨁◯◯◯ VERY LOW ^a^^,^^i^
Hospitalization with oxygen support or non-invasive ventilation follow up: 15 days	18 per 100	**18 per 100** (14 to 23)	**RR 0.98** (0.78 to 1.22)	1075 (2 RCTs)	⨁⨁◯◯ LOW ^a^
Discharge follow up: 15 days	45 per 100	**54 per 100** (48 to 61)	**RR 1.19** (1.05 to 1.34)	1075 (2 RCTs)	⨁⨁◯◯ LOW ^a^
Serious adverse events follow up: range 15 days to 28 days	27 per 100	**21 per 100** (17 to 25)	**RR 0.77** (0.63 to 0.94)	1296 (2 RCTs)	⨁⨁◯◯ LOW ^a^
Adverse events follow up: range 15 days to 28 days	37 per 100	**35 per 100** (30 to 41)	**RR 0.94** (0.81 to 1.10)	1296 (2 RCTs)	⨁⨁◯◯ LOW ^a^

Explanations

^a^. RoB: Wang et al. had some concerns of bias in the randomization process, and Beigel et al. had high risk of bias in the selection of the reported result.

^b^. Inconsistency: I2 = 43%.

^c^. Imprecision: 95%CI of effect is 0.39 to 1.28.

^d^. RoB: Wang et al. had some concerns of bias in the randomization process.

^e^. Imprecision: 95%CI of effect is 0.87 to 1.75.

^f^. RoB: Beigel et al. had high risk if bias in the selection of reported result.

^g^. Inconsistency: I2 = 60%.

^h^. Imprecision: 95%CI is 0.23 to 1.42.

^i^. Imprecision: 95%CI is 0.79 to 1.76.

**Table 3:** Summary of findings table for the comparison of remdesivir 5 days vs. remdesivir 10 days for hospitalized, severe COVID-19.

Patient or population: hospitalized, severe COVID-19						
Setting: Hospital						
Intervention: remdesivir for 5 days						
Comparison: remdesivir for 10 days						
Outcomes	Anticipated absolute effects* (95% CI)		Relative effect (95% CI)	No of participants (studies)		Certainty of the evidence (GRADE)
	Risk with remdesivir for 10 days	Risk with remdesivir for 5 days				
All-cause mortality follow up: 14 days	11 per 100	**8 per 100** (4 to 15)	**RR 0.75** (0.40 to 1.40)	397 (1 RCT)	⨁◯◯◯ VERY LOW ^a^^,^^b^	
Clinical improvement assessed with: Improvement of at least 2 points in a 7-point ordinal scale follow up: 14 days	54 per 100	**46 per 100** (38 to 55)	**HR 0.79** (0.61 to 1.01)	397 (1 RCT)	⨁⨁◯◯ LOW ^a^	
Recovery assessed with: Improvement from a baseline score of 2–5 to 6–7 in a 7-point ordinal scale follow up: 14 days	54 per 100	**47 per 100** (39 to 55)	**HR 0.81** (0.64 to 1.04)	397 (1 RCT)	⨁⨁◯◯ LOW ^a^	
Need for invasive ventilation follow up: 14 days	17 per 100	**8 per 100** (5 to 14)	**RR 0.48** (0.27 to 0.84)	397 (1 RCT)	⨁⨁◯◯ LOW ^a^	
Hospitalization without oxygen follow up: 14 days	8 per 100	**10 per 100** (5 to 19)	**RR 1.23** (0.66 to 2.31)	397 (1 RCT)	⨁◯◯◯ VERY LOW ^a^^,^^c^	
Hospitalization with oxygen support or non-invasive ventilation follow up: 14 days	12 per 100	**14 per 100** (8 to 23)	**RR 1.15** (0.69 to 1.91)	397 (1 RCT)	⨁◯◯◯ VERY LOW ^a^^,^^d^	
Discharge follow up: 14 days	52 per 100	**60 per 100** (50 to 72)	**RR 1.15** (0.96 to 1.37)	397 (1 RCT)	⨁⨁◯◯ LOW ^a^	
Serious adverse events follow up: 14 days	35 per 100	**21 per 100** (15 to 29)	**RR 0.61** (0.44 to 0.85)	397 (1 RCT)	⨁⨁◯◯ LOW ^a^	
Adverse events follow up: 14 days	74 per 100	**71 per 100** (63 to 79)	**RR 0.96** (0.85 to 1.08)	397 (1 RCT)	⨁⨁◯◯ LOW ^a^	

Explanations

^a^. RoB: Goldman et al. is at high risk of bias due to bias of measurement of the outcome.

^b^. Imprecision: 95%CI of effect is 0.40 to 1.40.

^c^. Imprecision: 95%CI of effect is 0.66 to 2.31.

^d^. Imprecision: 95%CI of effect is 0.69 to 1.91.

**Table 4:** Summary of findings table for the comparison remdesivir 5 days vs. standard of care for hospitalized, moderate COVID-19.

Patient or population: hospitalized, moderate COVID-19					
Setting: Hospital					
Intervention: remdesivir for 5 days					
Comparison: standard of care					
Outcomes	Anticipated absolute effects* (95% CI)		Relative effect (95% CI)	No of participants (studies)	Certainty of the evidence (GRADE)
	Risk with standard of care	Risk with remdesivir for 5 days			
All-cause mortality follow up: 11 days	2 per 100	**0 per 100** (0 to 0)	not estimable	391 (1 RCT)	⨁◯◯◯ VERY LOW ^a^^,^^b^^,^^c^
All-cause mortality follow up: 28 days	2 per 100	**1 per 100** (0 to 6)	**RR 0.52** (0.10 to 2.83)	391 (1 RCT)	⨁◯◯◯ VERY LOW ^a^^,^^b^^,^^d^
Clinical status assessed with: 7-point ordinal scale and proportional odds model follow up: 11 days	0 per 100	**0 per 100** (0 to 0)	**OR 1.65** (1.09 to 2.48)	391 (1 RCT)	⨁◯◯◯ VERY LOW ^a^^,^^b^^,^^e^
Clinical improvement assessed with: Improvement of at least 2 points from baseline on the 7-point ordinal scale follow up: 11 days	61 per 100	**70 per 100** (61 to 81)	**RR 1.16** (1.00 to 1.34)	391 (1 RCT)	⨁⨁◯◯ LOW ^a^^,^^b^
Recovery assessed with: Improvement from a baseline score 2–5 to a score 6–7 OR from baseline score 6 to score 7 in a 7-point ordinal scale follow up: 11 days	64 per 100	**74 per 100** (65 to 84)	**RR 1.15** (1.01 to 1.32)	391 (1 RCT)	⨁⨁◯◯ LOW ^a^^,^^b^
Need of invasive ventilation follow up: 11 days	2 per 100	**0 per 100** (0 to 0)	not estimable	391 (1 RCT)	⨁◯◯◯ VERY LOW ^a^^,^^b^^,^^c^
Hospitalization without oxygen follow up: 11 days	27 per 100	**23 per 100** (17 to 33)	**RR 0.87** (0.62 to 1.23)	391 (1 RCT)	⨁⨁◯◯ LOW ^a^^,^^b^
Hospitalization with oxygen support or non-invasive ventilation follow up: 11 days	9 per 100	**6 per 100** (3 to 13)	**RR 0.70** (0.35 to 1.41)	391 (1 RCT)	⨁◯◯◯ VERY LOW ^a^^,^^b^^,^^f^
Discharge follow up: 11 days	60 per 100	**70 per 100** (61 to 81)	**RR 1.17** (1.01 to 1.35)	391 (1 RCT)	⨁⨁◯◯ LOW ^a^^,^^b^
Serious adverse events follow up: 11 days	9 per 100	**5 per 100** (2 to 10)	**RR 0.52** (0.24 to 1.14)	391 (1 RCT)	⨁◯◯◯ VERY LOW ^a^^,^^b^^,^^g^
Adverse events follow up: 11 days	47 per 100	**51 per 100** (42 to 63)	**RR 1.10** (0.90 to 1.35)	391 (1 RCT)	⨁⨁◯◯ LOW ^a^^,^^b^

Explanations

^a^. RoB: Spinner et al. had some concerns of risk of bias due to bias of selection of the reported result.

^b^. Indirectness: Patients were hospitalized with SatO2>94% (no need of oxygen), described as moderate COVID-19.

^c^. Imprecision: RR is 0, and the upper 95%CI is infinite.

^d^. Imprecision: 95%CI of effect is 0.10 to 2.83.

^e^. Imprecision: 95%CI of effect is 1.09 to 2.48.

^f^. Imprecision: 95%CI of effect is 0.35 to 1.41.

^g^. Imprecision: 95%CI of effect is 0.24 to 1.14.

### Ongoing trials

S2 Table describes details of four ongoing remdesivir RCTs [27–33]. Further details of these RCTs can also be found in the S2 File.

## Discussion

In adult, hospitalized, RT-PCR confirmed COVID-19 patients with respiratory insufficiency or pneumonia, there were scarce data on efficacy and safety associated with the use of 10-day remdesivir regimens, or with the comparison of 5-day or 10-day regimens vs. standard of care. Two RCTs used a common treatment regimen and a true placebo control, and two RCTs compared two different doses of remdesivir, including one RCT with standard of care. Three trials were focused on severely ill COVID-19 patients, while one RCT was in mild to moderate COVID-19 patients. Remdesivir did not decrease all-cause mortality and need for invasive ventilation vs. placebo at 14 days but had fewer SAEs. Five-days of remdesivir decreased need for invasive ventilation and SAEs vs. 10-days of therapy. No differences in all-cause mortality or SAEs were seen among 5-days of 10-days of remdesivir and standard of care. Time to recovery was decreased by 4 days when remdesivir was compared to placebo, and by 0.8 days when patients were given 5-days of remdesivir vs. 10-days of therapy. Clinical improvement was higher with5-days of remdesivir vs. standard of care. The RCTs ranged from some concerns of bias to high risk of bias, and quality of evidence was low to very low for all outcomes and all comparisons.

The RCTs used different primary outcomes. The Wang et al. trial evaluated time to clinical improvement at 28 days, Beigel et al. trial evaluated time to recovery at 29 days and reported at 15 days, Goldman et al. evaluated clinical status at day 14, and Spinner et al. evaluated clinical status at day 11. Each of them used similar ordinal scale of disease severity. Furthermore, the four RCTs assessed the safety of this drug by measuring overall, specific and serious adverse events. The two-case series provided additional information about these outcomes as well.

While overall mortality is the most important final health outcome, it was not the primary endpoint of any of the available trials. Clinical severity is a relevant clinical outcome, but the ordinal scale used in these trials has not been validated before or appropriately analyzed. Indeed, there is not a correlation between clinical important differences and changes in scale scores in these trials. That may be because only the Beigel et al. and Spinner et al. trials used the proportional odds models and no trial used other methods such as the sliding dichotomy model. Serious adverse events, such as acute respiratory failure, acute kidney injury, and hypotension, are relevant as main metrics of safety.

Wang et al. RCT was stopped prematurely due to an excess of serious adverse events causing drug discontinuation, and Beigel et al. preliminarily reported their data at 15-days. While Wang et al. stated the use of a priori protocol that specified their stopping rule, there is no independent verification in their publicly available protocol. The demographic differences, with more remdesivir patients having hypertension, diabetes, or CAD, would be more likely to work against remdesivir efficacy. While more patients in the control group had been symptomatic for ≤10 days at the time of starting the intervention, this is not expected to cause more outcomes to occur. In Beigel et al., they changed their primary outcome twice [25] with no clear rationale for this alteration. Goldman et al. RCT also changed their primary outcome after the beginning of enrollment, and Spinner et al. trial reported their primary outcome at 11 days. In comparison to placebo, remdesivir did not significantly impact all-cause mortality, need of invasive ventilation, hospitalization without oxygen, hospitalization with oxygen or non-invasive ventilation, or treatment discontinuation. There was a significantly lower incidence of SAEs and higher proportion of discharged patients in RCTs comparing remdesivir to placebo. In comparison to 10-days of remdesivir therapy in Goldman et al., the 5-day regimen did not decrease mortality but decreased need for invasive ventilation and SAEs. AEs were similar between remdesivir and placebo arms and between the 5-day vs 10-day remdesivir arms. No differences in all-cause mortality, SAEs or AEs were seen among the 5-day, 10-day and standard of care arms in the Skinner et al. trial. There were substantial differences between the two-case series in the magnitude of their outcomes.

There were some differences in the scales used to assess outcomes across studies as shown in Table 1. Wang et al. and Grein et al. used the same 6-point ordinal scale, [15, 17] Beigel et al. used an 8-point scale, and Antinori et al used a 7-point scale. [16, 18]. Goldman et al. used a 7-point scale based on the 6-point scale of Wang et al. [19, 15] and Spinner et al. trial used a 7-point ordinal severity scale without further description [20]. These scales were strongly correlated, and that allowed us to formally group those scale categories in five in order to perform our meta-analyses for the two placebo-controlled RCTs [15, 16]; explanations are shown in S3 File. It is important to highlight that although these types of scales are based on a blueprint from WHO, neither of them has been fully validated as a disease severity index, and there is no current information about a more proper tool to assess the severity of COVID-19 [23].

Remdesivir is FDA approved and indicated for the treatment of hospitalized COVID-19 patients who are 12 years of age and older, and who weigh at least 40 kg. It should only be administered in a hospital or in a healthcare setting capable of providing acute care comparable to inpatient hospital care. We disagree that the currently available data is sufficiently strong to support its FDA approval, given that it is not possible to fully assess the balance of benefits to harms in COVID-19 infected patients. There is a risk that the benefits and harms of remdesivir will remain unknown if other remdesivir RCTs vs. placebo are stopped and substituted with trials where remdesivir becomes the standard of care and other experimental drugs are added onto remdesivir versus remdesivir alone. Patients may specifically ask for remdesivir therapy if they are not candidates for corticosteroid therapy and feel a failure to use an FDA approved option is a substandard practice.

Our systematic review has several strengths. We ran a recent and extensive systematic search in several engines and websites, and we did not restrict by language. We found commonalities across all studies: adult, hospitalized patients with COVID-19, and in particular patients with pneumonia and respiratory insufficiency. All six studies evaluated the same loading dose and a similar daily dose, albeit for different days of therapy, and two RCTs were compared to placebo. We also systematically searched for worldwide ongoing RCTs and ongoing systematic reviews in PROSPERO that can be found in S3 Table.

Some limitations can be highlighted. First, the number of RCTs was scarce, the reporting of the Beigel et al. trial is based on 15-day outcomes of the totality of recruited patients, and the Spinner et al. trial compared outcomes over11 days. Second, the Wang et al. RCT was stopped early because a higher proportion of adverse events leading to drug discontinuation was found in an unplanned interim analysis [15]. Third, our meta-analyses for primary outcomes and secondary outcomes were based only on two placebo-controlled RCTs and we used outcomes at similar time points of follow up and re-categorized heterogeneous ordinal outcome scales into five categories. Based on this, conclusions about mortality, need for invasive ventilation, and SAEs should be interpreted with caution. Whether it is the six- or seven-point scales used by investigators, these scales do not have an established minimum clinically important difference. Finally, three of the RCTs included patients given therapy within 10 to 15 days of when symptoms began. Remdesivir’s antiviral activity should be the highest during the first few days of active viral multiplication, as supported by a study performed in monkeys where early administration of remdesivir prevent progression to pneumonia after SARS-CoV-2 inoculation [8].

## Conclusions

There is paucity of adequately powered and fully reported RCTs evaluating efficacy and harms of remdesivir use in adult, hospitalized, COVID-19 patients. One RCT was stopped early without a clear description of the reasons, the largest trial (Beigel et al. ACTT-1), altered their primary endpoint twice and only reported 15-day outcomes, the Goldman et al. and Spinner et al. SIMPLE trials did not have a placebo arm, and the Spinner et al. SIMPLE trial outcomes were reported at 11 days. Conclusions about overall mortality, need for invasive ventilation, and SAEs from meta-analyses of two trials should be interpreted with caution. Several ongoing RCTs should be completed despite the FDA approval in order to determine remdesivir’s clinical efficacy and harm profile. Until stronger evidence emerges, we cannot conclude that remdesivir is efficacious for treating COVID-19.

## Supporting information

S1 File Pubmed search strategy: Long form. (PDF)

S2 File Description of ongoing RCTs evaluating remdesivir. (PDF)

S3 File Explanation of combination of categories of ordinal severity scales from RCTs. (PDF)

S1 Fig Effect of remdesivir vs. placebo on serious adverse events. (PDF)

S2 Fig Effect of remdesivir vs. placebo on hospitalized patients with no oxygen. (PDF)

S3 Fig Effect of remdesivir vs. placebo on hospitalized patients with oxygen support or non-invasive ventilation. (PDF)

S4 Fig Effect of remdesivir vs. placebo on discharged patients. (PDF)

S5 Fig Effect of remdesivir vs. placebo on treatment discontinuation. (PDF)

S6 Fig Effect of remdesivir vs. placebo on adverse events. (PDF)

S7 Fig Effect of remdesivir vs. placebo on anemia. (PDF)

S8 Fig Effect of remdesivir vs. placebo on elevated liver enzymes. (PDF)

S9 Fig Effect of remdesivir vs. placebo on hyperbilirubinemia. (PDF)

S10 Fig Effect of remdesivir vs. placebo on hypoalbuminemia. (PDF)

S11 Fig Effect of remdesivir vs. placebo on deep vein thrombosis. (PDF)

S12 Fig Effect of remdesivir vs. placebo on pulmonary embolism. (PDF)

S13 Fig Effect of remdesivir vs. placebo on renal impairment. (PDF)

S14 Fig Detailed and individual risk of bias of included randomized controlled trials. (PDF)

S15 Fig Risk of bias of included randomized controlled trials. (PDF)

S1 Table Summary of findings table of the comparison remdesivir 10 days vs. standard of care in moderate COVID-19 patients. (PDF)

S2 Table List of ongoing remdesivir RCTs from trial registries. (PDF)

S3 Table List of ongoing remdesivir systematic reviews from PROSPERO registry. (PDF)

S1 Checklist (DOCX)
