# Supplementary material for: Efficacy and harms of remdesivir for the treatment of COVID-19: A systematic review and meta-analysis
Source: PLoS One. 2020 Dec 10;15(12):e0243705. doi: 10.1371/journal.pone.0243705 (PMC7728272; doi:10.1371/journal.pone.0243705)
Supplement: S1 File — (PDF) [file pone.0243705.s001.pdf]

Appendix A. PubMed search strategy: Long form

("remdesivir"[Supplementary Concept] OR "remdesivir"[All Fields]) AND (("COVID-19"[All Fields] OR "COVID-2019"[All Fields] OR "severe acute respiratory syndrome coronavirus 2"[Supplementary Concept] OR "severe acute respiratory syndrome coronavirus 2"[All Fields] OR "2019-nCoV"[All Fields] OR "SARS-CoV-2"[All Fields] OR "2019nCoV"[All Fields] OR (("Wuhan"[All Fields] AND ("coronavirus"[MeSH Terms] OR "coronavirus"[All Fields])) AND (2019/12[PDAT] OR 2020[PDAT]))) OR ("coronavirus"[MeSH Terms] OR "coronavirus"[All Fields]) OR (("coronavirus"[MeSH Terms] OR "coronavirus"[All Fields]) AND ("disease"[MeSH Terms] OR "disease"[All Fields])) OR ("COVID-19"[Supplementary Concept] OR "COVID-19"[All Fields] OR "coronavirus disease 19"[All Fields]) OR ("severe acute respiratory syndrome"[MeSH Terms] OR ("severe"[All Fields] AND "acute"[All Fields] AND "respiratory"[All Fields] AND "syndrome"[All Fields]) OR "severe acute respiratory syndrome"[All Fields]) OR ("severe acute respiratory syndrome coronavirus 2"[Supplementary Concept] OR "severe acute respiratory syndrome coronavirus 2"[All Fields] OR "sars cov 2"[All Fields]))
