# Supplementary material for: Efficacy and harms of remdesivir for the treatment of COVID-19: A systematic review and meta-analysis
Source: PLoS One. 2020 Dec 10;15(12):e0243705. doi: 10.1371/journal.pone.0243705 (PMC7728272; doi:10.1371/journal.pone.0243705)
Supplement: S2 File — (PDF) [file pone.0243705.s002.pdf]

Appendix B. Description of Ongoing RCTs evaluating remdesivir

The WHO SOLIDARITY trial (ISRCTN83971151) compares the effectiveness of four treatments against standard of care in adult hospitalized COVID-19 patients. The primary outcome is all-cause mortality and the secondary outcomes are duration of hospital stay and time to first receiving intensive care or invasive ventilation. Participants are randomized to receive remdesivir or three other active treatments. We identified four ongoing SOLIDARITY clinical trial locations (Table 2). The Norwegian location (NOR) (NCT04321616)[25], the Iranian location (IRCT20200405046953N1)[24], the Canadian location (CATCO) (NCT04330690)[27] and the Spanish/Italian location[28] (EUCTR2020-001366-11).

The ACTT version from the European Union and United Kingdom[30] (EUCTR2020-001052-18) evaluates clinical efficacy of remdesivir compared to placebo for the treatment of COVID-19 in hospitalized adult patients. Their primary outcome is an 8-point ordinal scale at day 15. The DisCoVeRy trial[29] (NCT04315948) is a multi-center, adaptive, randomized, open clinical trial that evaluates safety and efficacy of treatments such as remdesivir and three active treatments compared with standard of care in 3100 adults hospitalized patients with PCR confirmed COVID-19. Its primary outcome is a 7-point ordinal severity scale at day 15.

Finally, the Fleming Directed COVID-19 Treatment Protocol[28] uses nuclear imaging to quantify measurements of isotopes over time due to changes in tissue resulting from metabolic and regional blood differences caused by COVID-19. This factorial 11-arm RCT planned to randomize 500 patients and measure the tissue improvement with the Fleming Method for Tissue and Vascular Differentiation and Metabolism (FMTVDM) at 72 hours after remdesivir and ten other active treatments.
