# Supplementary material for: Efficacy and harms of remdesivir for the treatment of COVID-19: A systematic review and meta-analysis
Source: PLoS One. 2020 Dec 10;15(12):e0243705. doi: 10.1371/journal.pone.0243705 (PMC7728272; doi:10.1371/journal.pone.0243705)
Supplement: S3 File — (PDF) [file pone.0243705.s003.pdf]

Appendix C. Explanation of combination of categories of ordinal severity scales from RCTs

We merged the 6-point severity scale used in Wang et al.[11] and the 8-point severity scale used in Beigel et al.[12] into 5 ordinal categories (outpatient; hospitalized, non-oxygen user; hospitalized, oxygen user non-invasive; hospitalized, invasive ventilation; and death). The first category is a merge of Wang et al. category 1, discharge alive, and Beigel et al. categories 1 and 2, not hospitalized without limitations and with limitations respectively. Hospitalized, non-oxygen user is a self-explanatory. Hospitalized oxygen non-invasive category merged Wang et al 3 and 4 and Beigel et al. 5 and 6 (see description of study). Finally, category hospitalized invasive ventilation corresponds to all patients with either mechanical ventilation or ECMO in category 5 in Wang et al or 6 in Beigel et al.
