# Supplementary figures and images for: Efficacy and harms of remdesivir for the treatment of COVID-19: A systematic review and meta-analysis
Source: PLoS One. 2020 Dec 10;15(12):e0243705. doi: 10.1371/journal.pone.0243705 (PMC7728272; doi:10.1371/journal.pone.0243705)

**Figure S1.** Effect of remdesivir vs. placebo on serious adverse events

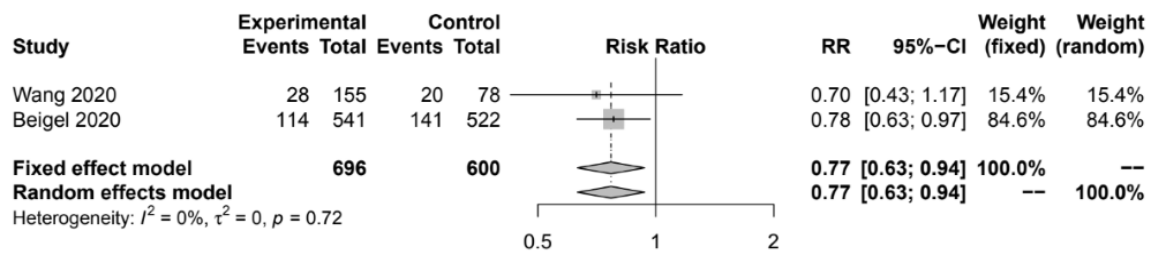

Supplement: S1 Fig — (PDF) [file pone.0243705.s004.pdf]

**Figure S2.** Effect of remdesivir vs. placebo on hospitalization with no oxygen

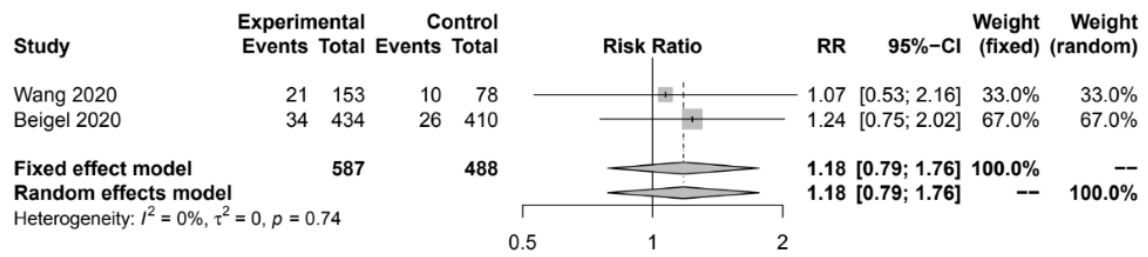

Supplement: S2 Fig — (PDF) [file pone.0243705.s005.pdf]

**Figure S4.** Effect of remdesivir vs. placebo on discharge

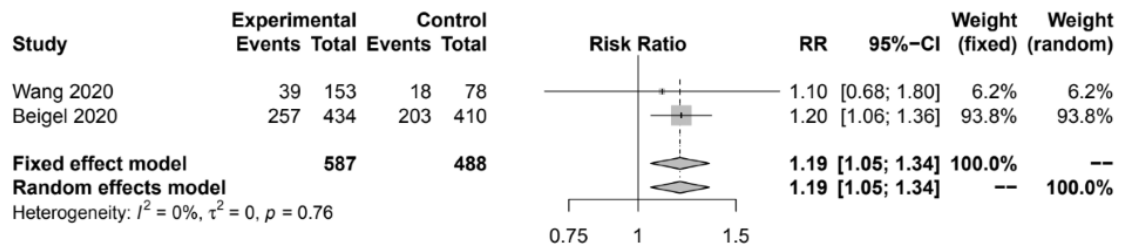

Supplement: S4 Fig — (PDF) [file pone.0243705.s007.pdf]

**Figure S5.** Effect of remdesivir vs. placebo on treatment discontinuation

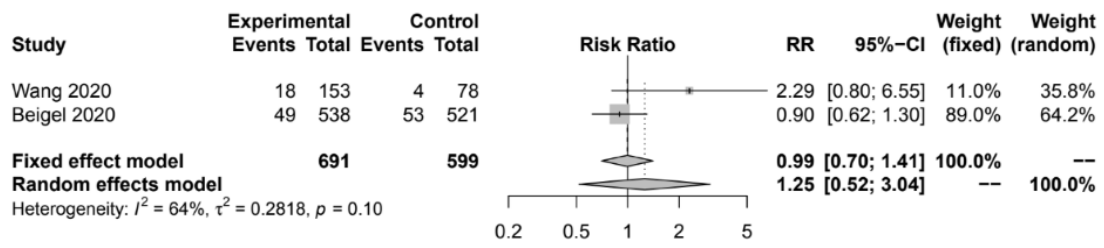

Supplement: S5 Fig — (PDF) [file pone.0243705.s008.pdf]

**Figure S6.** Effect of remdesivir vs. placebo on adverse events

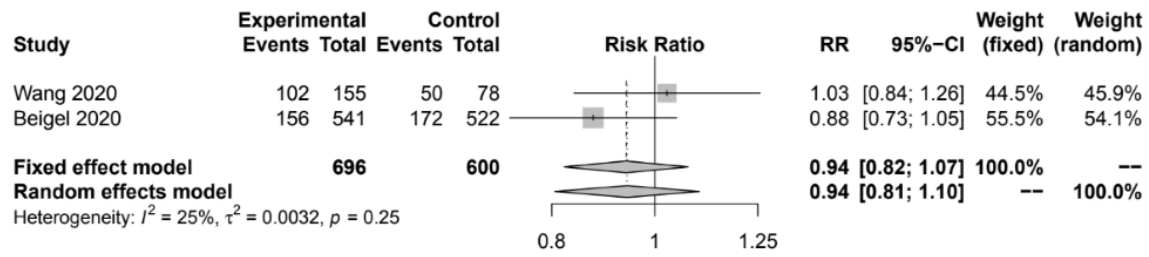

Supplement: S6 Fig — (PDF) [file pone.0243705.s009.pdf]

**Figure S7.** Effect of remdesivir vs. placebo on anemia

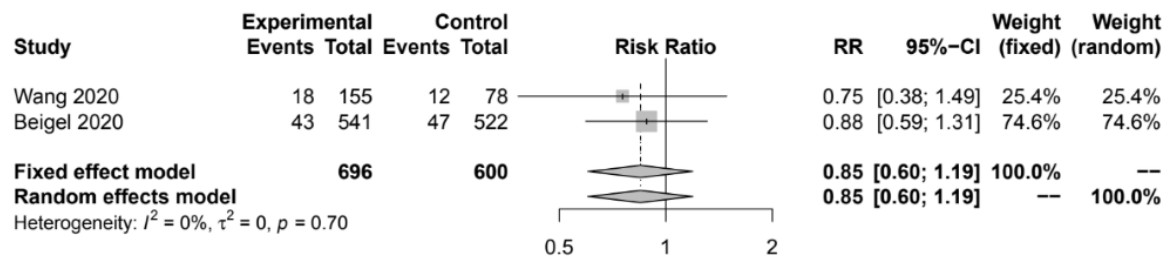

Supplement: S7 Fig — (PDF) [file pone.0243705.s010.pdf]

**Figure S8.** Effect of remdesivir vs. placebo on elevated liver enzymes

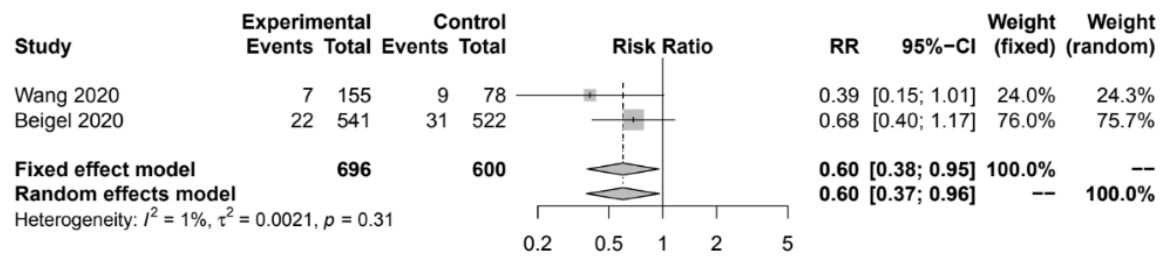

Supplement: S8 Fig — (PDF) [file pone.0243705.s011.pdf]

**Figure S9.** Effect of remdesivir vs. placebo on hyperbilirubinemia

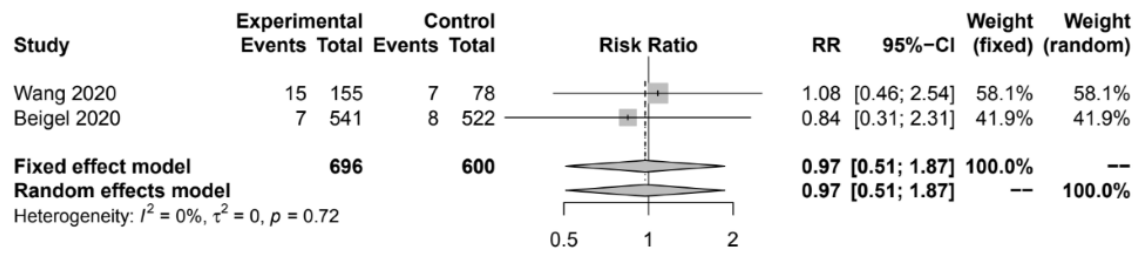

Supplement: S9 Fig — (PDF) [file pone.0243705.s012.pdf]

**Figure S10.** Effect of remdesivir vs. placebo on hypoalbuminemia

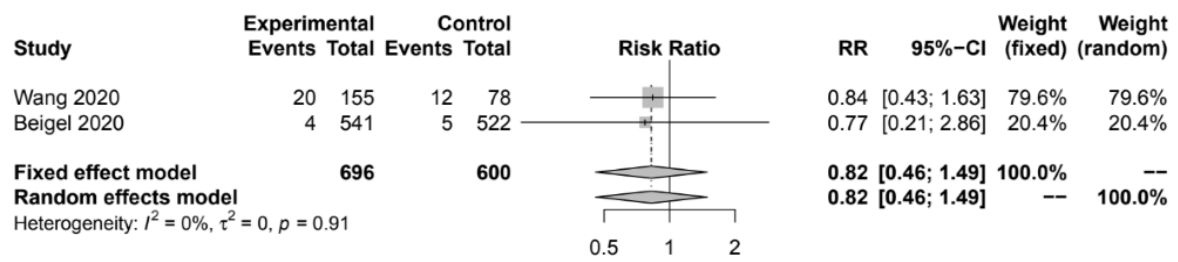

Supplement: S10 Fig — (PDF) [file pone.0243705.s013.pdf]

**Figure S11.** Effect of remdesivir vs. placebo on deep vein thrombosis

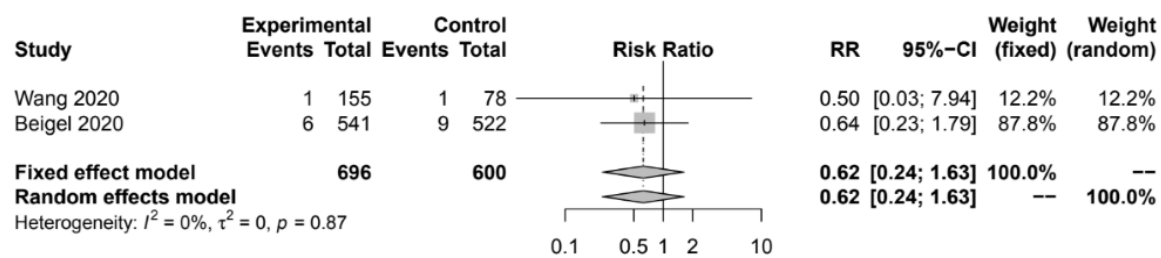

Supplement: S11 Fig — (PDF) [file pone.0243705.s014.pdf]

**Figure S12.** Effect of remdesivir vs. placebo on pulmonary embolism

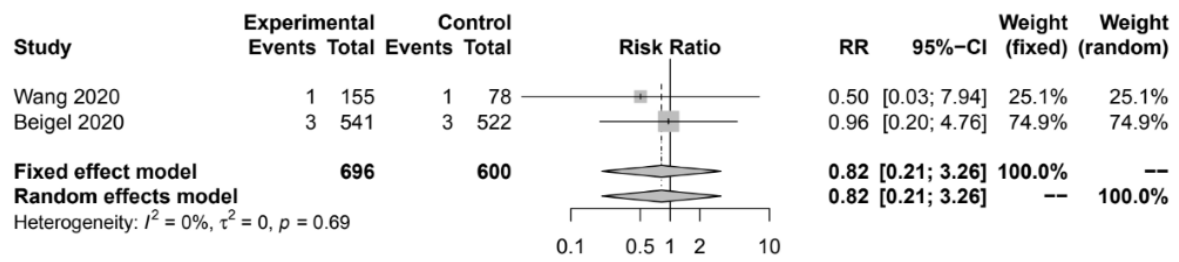

Supplement: S12 Fig — (PDF) [file pone.0243705.s015.pdf]

**Figure S13.** Effect of remdesivir vs. placebo on renal impairment

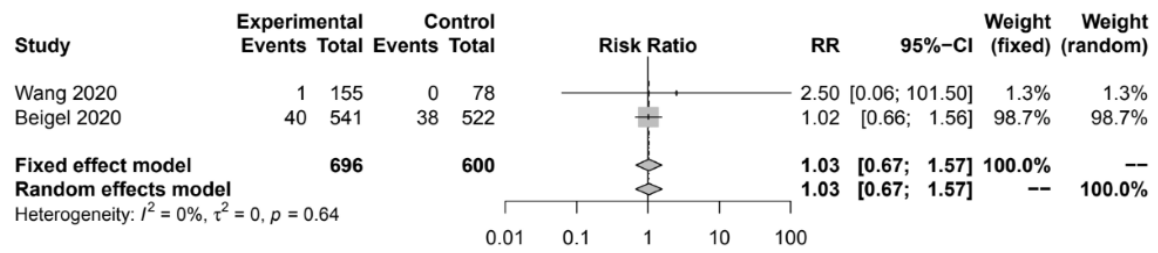

Supplement: S13 Fig — (PDF) [file pone.0243705.s016.pdf]

**Figure S15.** Risk of bias of included randomized controlled trials

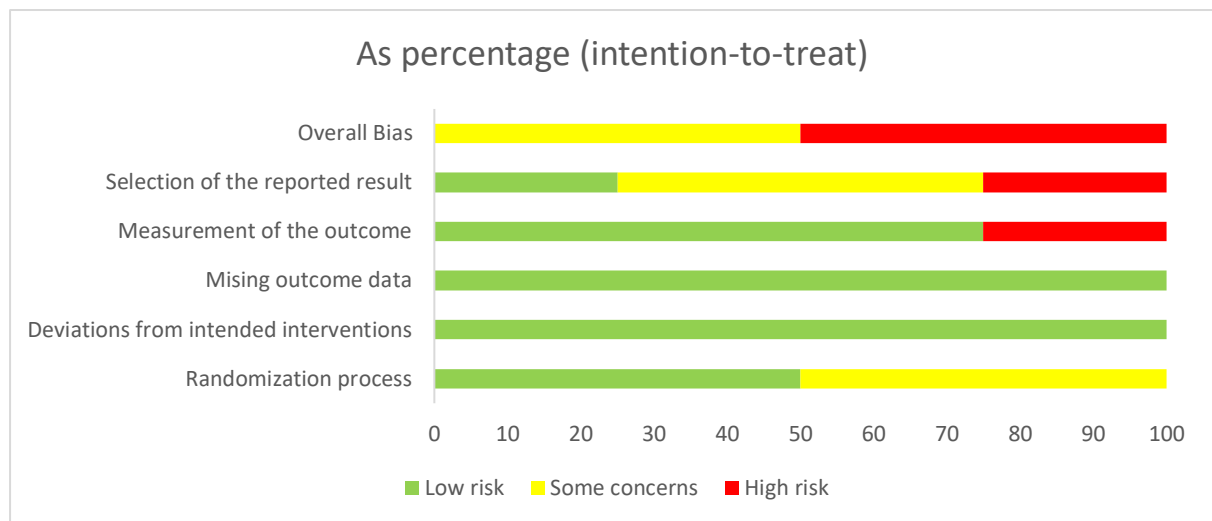

Supplement: S15 Fig — (PDF) [file pone.0243705.s018.pdf]
