# Supplementary material for: Efficacy and harms of remdesivir for the treatment of COVID-19: A systematic review and meta-analysis
Source: PLoS One. 2020 Dec 10;15(12):e0243705. doi: 10.1371/journal.pone.0243705 (PMC7728272; doi:10.1371/journal.pone.0243705)

Figure S3. Effect of remdesivir vs. placebo on hospitalization with oxygen support or non-invasive ventilation

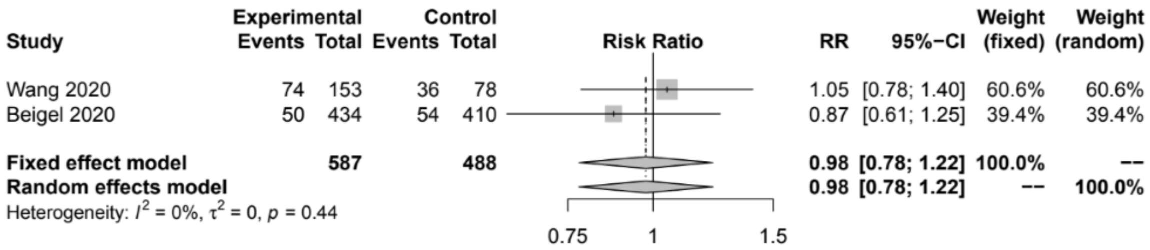

Supplement: S3 Fig — (PDF) [file pone.0243705.s006.pdf]
