# Supplementary material for: Efficacy and harms of remdesivir for the treatment of COVID-19: A systematic review and meta-analysis
Source: PLoS One. 2020 Dec 10;15(12):e0243705. doi: 10.1371/journal.pone.0243705 (PMC7728272; doi:10.1371/journal.pone.0243705)
Supplement: S14 Fig — (PDF) [file pone.0243705.s017.pdf]

Figure S14. Detailed and individual risk of bias of included randomized controlled trials

Studies with intention-to-treat	Unique ID	Study ID	Experimental	Comparator	Outcome	Weight	Randomization process	Deviations from intended interventions	Missing outcome data	Measurement of the outcome	Selection of the reported result	Overall
	1	Wang et al.	Remdesivir 10 da	Placebo	Time to clinical ir	1	?	+	+	+	+	!
	2	Beigel et al.	Remdesivir 10 da	Placebo	Time to recovery	1	+	+	+	+	-	-
	3	Goldman et al.	IV remdesivir 5 d	IV remdesivir 10	Day 14 clinical st	1	?	+	+	-	?	-
	4	Spinner et al.	Remdesivir 5 day	Standard of care	Clinical status	1	+	+	+	+	?	!

+	Low risk
?	Some concerns
-	High risk
