# Supplementary material for: Efficacy and harms of remdesivir for the treatment of COVID-19: A systematic review and meta-analysis
Source: PLoS One. 2020 Dec 10;15(12):e0243705. doi: 10.1371/journal.pone.0243705 (PMC7728272; doi:10.1371/journal.pone.0243705)
Supplement: S1 Table — (PDF) [file pone.0243705.s019.pdf]

S1 Table: Remdesivir for 10 days compared to standard of care for hospitalized, moderate COVID-19

Patient or population: hospitalized, moderate COVID-19
Setting: Hospital
Intervention: remdesivir for 10 days
Comparison: standard of care

Outcomes	Anticipated absolute effects* (95% CI)		Relative effect (95% CI)	№ of participants (studies)	Certainty of the evidence (GRADE)
	Risk with standard of care	Risk with remdesivir for 10 days			
All-cause mortality follow up: 11 days	2 per 100	**1 per 100** (0 to 6)	**RR 0.52** (0.10 to 2.80)	393 (1 RCT)	⨁◯◯◯ VERY LOW a,b,c
All-cause mortality follow up: 28 days	2 per 100	**2 per 100** (0 to 7)	**RR 0.78** (0.18 to 3.43)	393 (1 RCT)	⨁◯◯◯ VERY LOW a,b,d
Clinical status assessed with: 7-point ordinal scale and proportional odds model. follow up: 11 days	0 per 100	**0 per 100** (0 to 0)	**OR 1.31** (0.88 to 1.95)	393 (1 RCT)	⨁◯◯◯ VERY LOW a,b,e
Clinical improvement assessed with: Improvement of at least 2 points from baseline on the 7-point ordinal scale follow up: 11 days	61 per 100	**65 per 100** (56 to 76)	**RR 1.08** (0.93 to 1.26)	393 (1 RCT)	⨁⨁◯◯ LOW a,b
Recovery assessed with: Improvement from a baseline score 2-5 to a score 6-7 OR from baseline score 6 to score 7 in a 7-point ordinal scale follow up: 11 days	64 per 100	**68 per 100** (60 to 79)	**RR 1.07** (0.93 to 1.23)	393 (1 RCT)	⨁⨁◯◯ LOW a,b
Need of invasive ventilation follow up: 11 days	2 per 100	**1 per 100** (0 to 5)	**RR 0.26** (0.03 to 2.30)	393 (1 RCT)	⨁◯◯◯ VERY LOW a,b,f
Hospitalization without oxygen follow up: 11 days	27 per 100	**28 per 100** (20 to 38)	**RR 1.02** (0.74 to 1.41)	393 (1 RCT)	⨁⨁◯◯ LOW a,b
Hospitalization with oxygen support or non-invasive ventilation follow up: 11 days	9 per 100	**6 per 100** (3 to 13)	**RR 0.69** (0.34 to 1.40)	393 (1 RCT)	⨁◯◯◯ VERY LOW a,b,g
Discharge follow up: 11 days	60 per 100	**65 per 100** (56 to 76)	**RR 1.08** (0.93 to 1.26)	393 (1 RCT)	⨁⨁◯◯ LOW a,b
Serious adverse events follow up: 11 days	9 per 100	**5 per 100** (2 to 11)	**RR 0.58** (0.27 to 1.22)	393 (1 RCT)	⨁◯◯◯ VERY LOW a,b,h
Adverse events follow up: 11 days	47 per 100	**59 per 100** (48 to 71)	**RR 1.26** (1.04 to 1.52)	393 (1 RCT)	⨁⨁◯◯ LOW a,b

Explanations

a. RoB: Spinner et al. had some concerns of risk of bias due to bias of selection of the reported result.
b. Indirectness: Patients were hospitalized with SatO2>94% (no need of oxygen), described as moderate COVID-19.
c. Imprecision: 95%CI of effect is 0.10 to 2.80
d. Imprecision: 95%CI of effect is 0.18 to 3.43
e. Imprecision: 95%CI of effect is 0.88 to 1.95
f. Imprecision: 95%CI of effect is 0.03 to 2.30
g. Imprecision: 95%CI of effect is 0.34 to 1.40
h. Imprecision: 95%CI of effect is 0.27 to 1.22
