# Supplementary material for: Efficacy and harms of remdesivir for the treatment of COVID-19: A systematic review and meta-analysis
Source: PLoS One. 2020 Dec 10;15(12):e0243705. doi: 10.1371/journal.pone.0243705 (PMC7728272; doi:10.1371/journal.pone.0243705)
Supplement: S2 Table — (PDF) [file pone.0243705.s020.pdf]

Table S2. List of ongoing remdesivir RCTs from trial registries

TRIAL ID	Title	Country	Type of Patient; Remdesivir regimen	Comparators	Completion Date
**IRCT20200405046953N1 (SOLIDARITY)** [24]	Randomized trial of additional treatments for COVID-19 in hospitalized patients who are all receiving the local standard of care- Iranian SOLIDARITY multicentre trial	Iran	Hospitalized; daily infusion for 10 days (dose unspecified)	Standard of Care; Chloroquine or HCQ; Lopinavir/Ritonavir; Lopinavir/Ritonavir + IFN B-1a	Ongoing
**NCT04321616 (SOLIDARITY)** [25]	The (Norwegian) NOR Solidarity Multicenter Trial on the Efficacy of Different Anti-viral Drugs in SARS-CoV-2 Infected Patients	Norway	Hospitalized; ICU; 200mg intravenous loading dose, followed by 100mg once-daily maintenance dose for 10 days	Standard of Care; HCQ	August, 2020 (Ongoing)
**EUCTR2020-001366-11 (SOLIDARITY)** [26]	An international randomized trial of additional treatments for COVID-19 in hospitalized patients who are all receiving the local standard of care	Spain; Italy	Hospitalized; 100mg intravenous (duration unspecified)	HCQ; Chloroquine; Lopinavir/Ritonavir; IFN B-1a	Ongoing
**NCT04330690 (SOLIDARITY)** [27]	A Multi-centre, Adaptive, Randomized, Open-label, Controlled Clinical Trial of the Safety and Efficacy of Investigational Therapeutics for the Treatment of COVID-19 in	Canada	Hospitalized; 200mg intravenous loading dose, followed by 100mg once-daily maintenance dose for 10 days	Standard of Care; HCQ; Lopinavir/Ritonavir	March 18, 2023 (Ongoing)

	Hospitalized Patients (CATCO: Canadian Treatments for COVID-19), in Conjunction With the Public Health Emergency SOLIDARITY Trial				
**NCT04349410** [28]	The Fleming [FMTVDM] Directed CoVid-19 Treatment Protocol	United States	Hospitalized; 200mg intravenous loading dose, followed by 100mg once-daily maintenance dose for 10 days	HCQ + Azithromycin; HCQ + Doxycycline; HCQ + Clindamycin; HCQ + Clindamycin + Low-Dose Primaquine; HCQ + Clindamycin + High-Dose Primaquine; Tocilizumab; Methylprednisolone; IFN A-2b; Losartan; Convalescent Serum	October 11, 2020 (Ongoing)
**NCT04315948 (DisCoVeRy)** [29]	Multi-centre, Adaptive, Randomized Trial of the Safety and Efficacy of Treatments of COVID-19 in Hospitalized Adults	France; Luxembourg	Mechanical Ventilation; 200mg intravenous loading dose, followed by 100mg once-daily maintenance dose for 10 days	Standard of Care; HCQ; Lopinavir/Ritonavir; Lopinavir/Ritonavir + IFN B-1a	March, 2023 (Ongoing)
**EUCTR2020-001052-18 (ACTT UK-EU)** [30]	A Multicenter, Adaptive, Randomized Blinded Controlled Trial of the Safety and Efficacy of Investigational Therapeutics for the Treatment of COVID-19 in	Denmark; UK; Greece; Spain	Hospitalized; Mechanical Ventilation; 100 to 200mg intravenous (duration unspecified)	Placebo	Ongoing

	Hospitalized Adults - Version for European Union/United Kingdom Sites				
