# Supplementary material for: Efficacy and harms of remdesivir for the treatment of COVID-19: A systematic review and meta-analysis
Source: PLoS One. 2020 Dec 10;15(12):e0243705. doi: 10.1371/journal.pone.0243705 (PMC7728272; doi:10.1371/journal.pone.0243705)
Supplement: S3 Table — (PDF) [file pone.0243705.s021.pdf]

Table S3. List of ongoing remdesivir systematic reviews from PROSPERO registry

PROSPERO ID	Title	Country	Type of Studies	Type of patient	Comparator(s)
**CRD42020180084**	A systematic review and meta-analysis of the efficacy of remdesivir and favipiravir for the treatment of COVID-19 infections	Kuwait, Saudi Arabia	RCT and non RCT	Adult and geriatric patients hospitalized for COVID-19 infections	Supportive and standard treatments options
**CRD42020183707**	A systematic review of the effectiveness of remdesivir for the treatment of patients with severe COVID-19	Indonesia	RCT and non RCT	Patients with severe positive or confirmed cases of COVID-19	Other Western medical therapies, supportive care, or other therapeutic methods
**CRD42020181509**	Efficacy and safety of Remdesivir for the treatment of patients with COVID-19: a systematic review and meta-analysis	USA	RCT and non RCT	Patients of all ages with a diagnosis of COVID-19/SARS-CoV-2.	Placebo or symptomatic management
**CRD42020177953**	Efficacy of remdesivir versus placebo for the treatment of coronavirus diseases 2019 (COVID-19): A protocol for systematic review and meta-analysis of randomized controlled trials.	Ethiopia	RCT	participants diagnosed with COVID-19 and with or without other co morbid conditions.	Placebo / standard of care
**CRD42020184077**	Evidence of efficacy and safety of remdesivir for the treatment of COVID-19: a protocol for a rapid systematic	China	RCT and non RCT	Patients diagnosed with COVID-19 regardless of sex, age, and race.	Placebo, other drugs or no intervention

	review and meta-analysis				
**CRD42020183384**	Remdesivir for COVID-19: A living systematic review protocol	Spain	RCT and non RCT	participants with COVID-19, as defined by the authors of the trials	Placebo or no treatment
**CRD42020168406**	Remdesivir for novel coronavirus (2019-nCoV) pneumonia: a systematic review and meta-analysis of randomised controlled trials	China	RCT	Patients diagnosed with pneumonia caused by new coronavirus infection (2019-nCoV).	Other Western medical therapies, supportive care or other therapeutic methods
